# Brain Donation Decisions as Disease Specific Behaviors: An Elucidation of the Donation Process in the Context of Essential Tremor

**DOI:** 10.5334/tohm.704

**Published:** 2022-08-23

**Authors:** Daniella Iglesias-Hernandez, Diane Berry, Nora Hernandez, Elan D. Louis

**Affiliations:** 1 Department of Neurology, University of Texas Southwestern Medical Center, Dallas, TX, US

**Keywords:** Brain donation, brain banks, brain repository, essential tremor, neurodegeneration

## Abstract

Brain donation is a challenging process, comprising four sequential stages: (1) the brain donation decision, (2) pre-mortem arrangements and follow up, (3) specimen collection and (4) tissue processing. It is important to understand the factors that are pertinent to each stage. Currently, there is extensive information on factors that involve donor’s personal and cultural backgrounds and how these could affect the process. However, little is known about *disease-specific factors* that influence the process. The Essential Tremor Centralized Brain Repository was established in 2003, and after nearly 20 years of collecting essential tremor (ET) brain tissue, we are well-positioned to discuss the brain donation process from a disease-specific standpoint. In the current manuscript, we discuss ET disease-specific factors that influence the first two stages of the brain donation process. We center our discussion around three points: (1) factors that influence the patient’s decision to donate, (2) the involvement of next of kin in the donation, and (3) the rationale for enrolling patients prospectively and evaluating them longitudinally before the anatomical gift takes place. This discussion shares our understanding of the background from which our repository operates and may be of value for other brain banks that study similar neurodegenerative diseases.

## Introduction

Anatomical and neuropathological studies of the brain have advanced considerably over time. What started as theoretical descriptions of brain tissue in ancient Greece, transformed progressively to rare brain collections in the 18^th^ century [1,2]. During the 1960s, the modern concept of *the brain repository*, a tissue- and disease-oriented archive with specific goals, was established [3,4]. Because of these repositories, we now have detailed descriptions of the underlying neuropathological changes in diseases such as Alzheimer’s disease and essential tremor (ET) [5,6]. It is important to note that these efforts rely heavily on the success of brain donations.

The brain donation process has evolved through the years and now incorporates multiple legal regulations to protect patients (e.g. donor’s rights and written consent) as well as a specific sequence of steps required to bequest such an anatomical gift [7]. These steps differ somewhat by repository, but generally involve four main stages: (1) the brain donation decision, (2) pre-mortem arrangements and clinical follow up, (3) specimen collection and (4) tissue processing.

The literature provides detailed accounts of each stage, but certain gaps in knowledge remain. First, researchers have investigated the factors that influence the likelihood that an individual will decide to donate their brain [8,9,10,11,12,13,14]. However, most research focuses on cultural and personal differences that influence this decision, and rarely considers disease-specific motivations. Second, some research has focused on how brain banks collect data prospectively and plan to harvest brain tissue in the future; however, little has been written about the rationale for such approaches [15,16]. That is, brain repositories seldom explain why they decide to follow participants years before the actual donation or, on the contrary, only chose to approach the donor’s family at the time of death [17,18].

In sum, the lengthy list of current publications does not capture all of the subtleties involved in each of the four stages of brain banking. In particular, there is little attention paid to the fact that the decision to donate and the pre-mortem arrangements and follow-up *may be influenced by the actual disease the repositories are trying to study*. In the world of brain donation, fashioning the appeal that an organization uses when approaching a particular donor or donor population is challenging. Having an understanding of both personal history and how the specific disease that afflicts patients would be of beneficial. One size does not fit all.

Analyzing these stages from the point of view of specific diseases could be useful when structuring and establishing specific brain repositories and could provide a better understanding of the background from which banks operate. Nowadays, we require that level of specificity in response to the high specialization of brain donations for numerous target conditions.

The Essential Tremor Centralized Brain Repository (ETCBR) was established in 2003. After nearly 20 years of banking brain tissue for ET, we are in an excellent position to discuss the donation process from a disease-specific standpoint [19]. In this manuscript, we discuss ET disease-specific factors that affect the first two stages of the brain donation process. We center our discussion around three points: (1) factors that influence the decision to donate, (2) involvement of family in the donation, and (3) rationale for enrolling participants prospectively and evaluating them longitudinally before the anatomical gift takes place.

### 1. The brain donation decision

For each repository, the consent to harvest is the first step towards a brain donation. This step is often difficult for patients, given the delicate end of life discussion involved. Moreover, these discussions may require a sensitivity to cultural influences and constraints operating on potential donors and their families. Currently, patients have multiple online resources available to assist them in exploring their options and better understand the positive impact that their decision can have on the future of others. Despite these tools, helping patients to arrive at a decision can be challenging; hence, an effective communication between the research team and potential donor is essential. During the ensuing conversations, it is important to keep the specifics of each patient’s disease and medical status in mind. Understanding the patients’ unique experiences shows honest empathy and provides the patient with the sense that the research team is sensitive to the specific struggles the patient faces, as they decide whether to pursue a brain donation. In this section, we explore the unique aspects of ET that may affect the donor’s decision to become part of the ETCBR.

#### A. The realities of ET

##### The therapeutic conditions

There are specific aspects of ET patients’ experiences that might influence their decisions to donate to a brain repository. First, although ET is one of the most prevalent movement disorders, the effectiveness of its treatment is both severely limited and inconsistent from case to case [20,21]. Providers often have the unpleasant task of teaching patients to keep their expectations of the success of attempts at tremor control low. This simply reflects the state of an ET patient’s reality: the likely effectiveness of front line medications for ET is quite low. For example, propranolol and primidone only provide between 40% to 50% tremor improvement, and patients are typically encouraged to be satisfied with this outcome [22]. As a result, patients often choose to discontinue pharmacological treatment and are lost to follow-up [23,24,25]. To make matters more complicated, not all ET patients are eligible for or want to opt for surgical treatments and their potential positive outcomes [23]. Thus, the ET community is underserved when it comes to effective treatment options [26]. One donor aptly remarked as follows: “Nothing [no treatment] is working for me; [I] hope you can do something with my brain to find a cure.”

Brain donors frequently discuss the above issues with research team members during enrollment in the ETCBR. They often share stories of unsatisfying experiences with multiple tremor medications. As a result, by the time patients come to the ETCBR, they often have given up pharmacological treatment for tremor control. Interestingly, the movement away from treatment we witness coexists with the hope of a cure for ET in the future. Patients are motivated by the lack of effective available medications and decide to contribute to the research through the brain donation. They believe the act can help in the mapping of crucial changes that occur in the brain due to ET, and this might pave the way towards novel and improved treatments. One donor noted, “I am so glad you are doing research on this” [ET]; perhaps you can find what is wrong with my brain and help others if not me.” Another remarked, “Yes, you can take my brain for research. I am happy to contribute to finding a cure or a treatment to stop the tremors.” Yet another told us, “My tremors are severe; I cannot do anything with my hands. I was searching in the internet and saw your study on essential tremor and the brain bank. I am glad you are doing this; you might find a treatment that could help prevent this from happening to others. I want to become a brain donor. I want to donate my brain after I die.”

##### Attitudes towards essential tremor and minimization a coping mechanism

A second reason that might increase the likelihood of brain donation in ET patients is directly linked to how others perceive and judge their disease, (e.g. health care providers and general public), and the coping mechanisms this community adopts to have a functional life.

Many ET patients perceive that health care providers do not take their motor manifestations as seriously as those associated with other movement disorders (e.g., Parkinson’s disease [PD], spinocerebellar ataxias). In fact, patients report that they often hear from their providers that ET is “only action tremor” and not a life-threatening condition, or “at least is not PD”. Similarly, the general public underestimates ET and more importantly, is barely aware of the disease [27,28]. There is even on some level a denial that the patient has a disease; this often results in uncomfortable and embarrassing remarks directed towards ET patients, such as “are you shaking because you are too nervous?” [29].

Nevertheless, the attitudes previously described are at odds with certain realities about ET [27,28]. For example, ET patients may actually experience more hours of tremor in a given day than do patients with PD [30]. Moreover, ET patients suffer from social anxiety, depression and other mood related disorders [31]. Feelings of embarrassment and inadequacy also plague this population [32]. Shame and fear of negative evaluations from peers are a focus of current research, and the psychological repercussions for patients are not fully known [33]. Despite the features described above and the possibility of disability due to severe tremor [34], ET patients still face these judgements that contradict the complexity of their reality, and as a result, they are forced to adopt minimization of tremor as a coping mechanism.

In the day-to-day operation of our brain bank, a comment frequently heard from donors is “It’s just tremor, I can live with it”. Inevitably, they downplay their motor manifestations as they modify their lives to accommodate those symptoms. Only when probed further are patients willing to admit the extent to which ET has changed their lives: e.g., that the shaking prevents them from eating in public or participating in their favorite activities (e.g. sculpting, photography), and even resulting in early retirement. In a recent paper, the authors noted that patients referred to their ET as “a nuisance but not a death sentence” and they strived to “stay positive and learn to fight in different ways” [29].

However, adopting minimization as a coping mechanism does not mean the ET community is oblivious to the repercussions of the disease. As a result, they often wish that their family members will not develop and suffer from tremor. If anything, noticing the symptoms in their grandchildren as well as the perceived lack of empathy from both health care providers and the general public gives these patients a special motivation to collaborate with research studies. The experience influences the brain donation decision, and they opt for it, because “It’s a gift for future generations” and they find this might help change judgements about ET “through more knowledge.” One donor remarked to us, “I remember my grandmother had very severe tremor in both her hands before she died. But I was too young, so I do not remember many details. My mother had it too, and at the end she could not feed herself or do anything with her hands; she was very disabled. I have had symptoms for years now and my doctor confirmed that I also have essential tremor. One of my two daughters have [sic] started with some mild tremor in her hands… I want to become a donor to help; your research might find out why this is happening in our family.” A donor told us: “I can see it in my 7-year-old grandson: his hands shake. I hope you can find a cure. I do not want for him the stigma and disability this disease carries.”

##### Information about ET has not permeated to the level of treating physicians

There has been considerable academic progress over the past decade. However, a third issue stems from the fact that the new information on ET has not completely permeated to general neurologists and primary care physicians (PCP) in the community. The concept of ET itself has changed. Earlier definitions considered ET to be a mono-symptomatic movement disorder. More recent views recognize its heterogeneity; there is an array of motor and non-motor features [35]. An area of scholarship centers on defining the specific set of manifestations that characterize ET. For example, cognitive decline often accompanies ET, and there is a discussion as to whether it is caused by ET, or merely correlated with it, due to age [36,37,38]. Action tremor (e.g. kinetic and postural tremor) is often described in the literature as the cardinal and sometimes only feature of ET [39]. However, ET is frequently characterized by multiple motor features that may or may not include severe action tremor [40]. For example, ET can present as kinetic tremor that coexists with intention and rest tremor in a given patient [41,42]. At the same time, as the disease progresses, other patients might experience Parkinsonism or dystonic posturing layered on top of the baseline ET features [43,44]. As a result, new terms such as “ET plus” have been introduced to account for these intricate phenotypes. From this mélange of new information has ensued complex academic debates, but these have not necessarily filtered down to general neurologists, general practitioners and patients themselves [45,46,47].

An additional reason for PCPs to have limited access to up-to-date information involves the rapid pace of new findings in clinical description, neuropathology findings and potential treatment of ET [48,49]. All of the above-mentioned factors, together, compromise the knowledge of treating physicians and the information or lack thereof they share with patients, resulting in frequent misdiagnosis [50].

For ET patients, these issues represent a real roadblock to receiving health care that meets their needs. Providers with inaccurate information are unable to answer questions posed by patients, which defeats the purpose of seeing a physician for tremor control and counseling. Furthermore, the physician’s inability to explain subtle changes in the patient’s symptoms can be a frustrating experience for the ET community, many of whom cease seeking medical counsel and care for their tremor. This has repercussions on the ETCBR, as many donors do not regularly see a neurologist. In turn, they seek to be involved in research as a way of answering questions the health care system does not fully address. They opt for a radical decision such as donating their brain in order to help change the narrative of ET.

#### B. Additional factors that contribute to brain donation in ET

For each potential ETCBR donor, there are additional factors that affect the likelihood of a decision to donate. In general, the brain donation literature describes the following aspects as positive factors that may increase the likelihood: plans for cremation as a funeral arrangement, support from family members, and having access to a clear explanation of how the donation takes place [51,52]. On the other hand, data also show that families that do not see the brain donation decision as an individual choice, that fear financial gain from the research team, and who are of African American or Asian ethnicity are all less likely to complete brain donations [51].

In our experience, participants who have exposure to previous brain donations in their families or are acquainted with people who are themselves brain donors tend to have a positive attitude towards the process. For example, some participants enroll because their parents completed a brain donation with our program and that was a powerful motivation for them. Similarly, several participants agree to the brain donation because their siblings, cousins or close friends are part of the study already. Therefore, the familial (i.e., highly genetic) nature of ET [53] seems to be an important factor in motivating ET patients and it would be beneficial to further explore this factor and its potential influence on the likelihood of brain donation. A donor told us, “My mother was a brain donor in your essential tremor study. Now after years of noticing my hand tremor worsen, my doctor confirmed that I have essential tremor too, so I have decided to become a brain donor. My two cousins also have tremor, and they will contact you to enroll in your study. Because it runs in our family and we have seen the degree of disability the tremors cause, we all want to help your research, for the sake of our family’s future and others’ who are suffering with this same disease.”

### 2. Donor’s family and their involvement in the brain donation

In the early 1900s, obtaining consents for brain donations was not a standardized procedure. Dissections occurred either *in lieu* of clear permission from the donor or without any type of agreement from the patients. Julius Hallervorden, a physician who harvested dozens of brains in collaboration with the Nazi regime, used “euthanasia” in vulnerable populations [54]; this case called attention to numerous procedural and regulatory deficits. In 1968, the USA passed the Uniform Anatomical Gift Act (UAGA) [55]. Due to these regulations, family members, and specifically the next of kin, are now required to be formally involved in these decisions, and their involvement has become invaluable. Without their efforts, harvesting brains would be more difficult and more limited geographically as brain donors sometimes reside far away from the physical repositories. Nowadays brain banks can extend their reach and enroll participants with the understanding that family members will assist in the final hours. For example, a donor who was an active advocate for ET and who lived in New York City died during the pandemic. Her daughter lived abroad and managed to travel the same day her mother died, but upon arrival she could not find where paramedics had taken the body. The city’s health system was strained due to the number of deaths caused by the pandemic. She underwent the most horrifying hours traveling to different medical centers until she located her mother’s body, listed as stored in a refrigerated container stationed in one of the boroughs. She immediately contacted us and helped us coordinate the brain procurement before it was too late. She said she would never forgive herself if she did not do this for her mother, whose life was so altered by the disease.

Depending on the repository, the role of the next of kin varies. For brain banks that follow participants years prior to their passing, their collaboration is supportive, as they fulfill a longstanding and pre-established brain donation wish from their loved ones. In the case of brain banks in which contact with the family occurs at the time of death, the next of kin may carry the weight of the brain donation decision without a sense of what their family member would have wanted. In this section, we explore the motivation of the next of kin to play their important role in the ETCBR.

#### A. Next of kin perception of ET

One motivation behind the next of kin’s involvement in the brain donation process could be their perception of the disease. Disease perception is a complex process in which dimensions of the disease (e.g. severity, suffering, and prognosis) are interpreted according to the observer’s socio-economic status, race, previous experience, and level of education. Patients, caregivers, and health care providers may have differing perceptions of a specific disease even though they all observe the same progression [56,57]. Specifically, family and friends tend to overestimate the suffering experienced by ET patients as compared to the patient’s own ratings [57]. Moreover, ET patients experience a myriad of psychological symptoms such as anxiety, depression, and fear of social events due to anticipation of poor performance, and the next of kin may also account for these factors when rating suffering [33,58].

The above-mentioned discrepancy between patient and family perceptions may reflect patient habituation; years of living with the same symptoms may actually modify the perception of the burden experienced. Furthermore, a recent publication established that there is no correlation between ET patients’ self-report of demoralization and objective measures of tremor severity (e.g. greater tremor severity does not seem to be associated with more demoralizing feelings), [59] despite the fact that patients do perceive that tremor worsens over time [60].

In the ETCBR, the sum of these factors provides the next of kin with a unique perspective on the repercussions of ET. From tremor severity to feelings of embarrassment, they perceive the array of symptoms affecting their loved ones day in and day out. In some cases, family members are the ones encouraging the brain donation. In many others, understanding the different dimensions of ET allows them to commit to their loved one’s wishes and see them fulfilled. Nevertheless, the complexity of next of kin’s comprehension warrants closer examination as it provides a counterpoint to the patient’s self-report. Evaluating other dimensions of how next of kin perceive ET might be beneficial.

#### B. Grief management in ET

The next of kin may also be motivated to participate in the brain donation process as a means of coping with grief. It is well known that the loss of cognitive function or mobility due to chronic disease in a loved one can affect the way family members grieve [61,62]. Specifically, in the case of Alzheimer’s disease, family members often experience ambiguous loss prior to the patient’s death; the patient is perceived as physically present but psychologically absent [63]. Furthermore, anticipatory grief is common in the families of dementia patients [64].

Making sense of the course of these degenerative diseases is difficult, and acts such as organ donation can help patients find meaning in their passing. Similarly, caregivers involved in the advance directive may find a sense of purpose in participating in their loved ones’ wishes. For example, a recent study reported that family members who completed whole body donations of a loved one to benefit medical schools, were more likely to find closure and resolution of their grief [64].

The literature further describes additional strategies to manage complicated grief. In the case of Alzheimer’s disease, delivering knowledge about the disease and related dementias can aid in conflict resolution and result in significant improvement in caregiver sadness, guilt and longing when combined with group-based programs [65]. Similarly, disease related education could help parents of children with cystic fibrosis overcome unresolved grief [66].

As part of the longitudinal follow up in the ETCBR, donors periodically complete thorough videotaped neurological evaluations and cognitive assessments. Often, they experience physical disabilities or marked cognitive decline, making the cooperation of next of kin crucial for the successful completion of these tasks. The next of kin later facilitates the brain donation, and, in their grief, carries out the plans made by the donor. This provides a remarkable opportunity for closure, and family members often note that it is a relief to “finish what my father/mother started”. As ET is associated with greater cognitive decline and higher rates of conversion to dementia than controls [36,38], it is not surprising that we have observed a response toward brain donation in the families of ET patients paralleling that observed for families of Alzheimer’s patients.

Nevertheless, attitudes towards acquiring more information about ET do not seem comparable with other diseases such as Alzheimer’s disease or cystic fibrosis. At the time of death, the ETCBR invites family members to discuss neuropathology findings and autopsy reports with the research team. We actively recommend these sessions but we leave to their discretion if and when to contact us. Surprisingly, a small proportion of families reach out to access the reports of their loved ones. The observation suggests that ET family members do view the brain donation as a key part of overcoming their grief, but obtaining more information about the disease itself (e.g. diagnostic confirmation) does not seem to be an important issue. The ET diagnosis is a clinical one and in the absence of postmortem-based diagnostic criteria limit the diagnostic yield of the postmortem examination as confirmatory of ET.

#### C. Caregiver’s burden in ET

Caregiver burden is a very common phenomenon that occurs in many diseases, and that may also influence the next of kin involvement in the ET brain donation [67]. This multidimensional construct includes the struggles faced by the caregiver, and can have a wide array of manifestations (e.g. depression, sleep disorders, financial strain, negative impact on physical health, and self-neglect) [67,68]. In addition, the burden varies, with individual differences of caregivers and the characteristics of the disease their family member faces [69]. In the case of dementia, the phenomenon is usually associated with intensity of the responsibility and the number of hours a day the family member takes care of the patient [70]. Additionally, dementia caregivers are at more risk of experiencing decline in their physical and mental health due to the stress related to the responsibilities they assume [71].

In ET, caregiver burden has specific characteristics as well. First, family members provide care to ET patients, which usually involves offering support with some motor activities (e.g. cooking, writing) [72]. However, the embarrassment experienced by ET patients due to their action tremor is often associated with more caregiver burden, and caregivers can play an active role in providing reassurance [32]. Furthermore, there is an additional emotional layer to ET caregivers, as they walk the fine line between assisting with activities of daily living while also promoting independence and reducing embarrassment [73].

In our experience, the dynamic of ET caregivers and patients lends additional meaning to the research interviews and the brain donation. Family members might view these two activities as tools to reduce embarrassment and reaffirm independence in their loved ones. The collaboration with the research team reflects their respect for the patient’s wishes, and their involvement allows the caregiver to ensure their family member completes their contribution to science.

### 3. Data collection prior to the donation

Organ repositories, especially brain banks, tend to work in an expedited manner and there are two approaches to banking brain tissue and collecting relevant data - either they accept anatomical gifts after a brief screening process at the time of death and collect necessary clinical information retrospectively through medical records, or their donors enroll years prior to the time of death and give advance directives for family and friends. This latter approach results in careful planning of the brain donation as well as prospective collection of detailed clinical data [74,75,76]. In the ETCBR, prospective, longitudinal follow-up is the cornerstone for establishing clinical-pathological correlations. In this section, we discuss the different elements that justify the decision to follow our participants prospectively.

#### A. Misdiagnosis of ET and repercussions on the enrollment process

ET is frequently misdiagnosed in the outpatient clinic [50]. The clinical phenomenology is subtle, and too often primary care providers and general neurologists assign the diagnosis without taking into account more nuanced considerations [40]. A recent study documented that patients with conditions such as dystonia, PD, and physiological tremor can be mis-labeled as ET in outpatient settings [50]. Furthermore, some patients tend to self-report their shaking as ET, although approximately 30% of individuals tend to misdiagnose their movement disorder regardless of their level of education [77].

During the nearly 20 years that the ETCBR has been active, on multiple occasions we have received calls from families interested in donating the brain of their decedent because they suffered from long standing ET. However, the decedent never consulted a health care provider regarding their tremor and self-report was the foundation for the ET diagnosis. Due to the time sensitive nature of a brain donation, confirming a diagnosis of ET before accepting a donation is extremely difficult, especially when most potential donors lack previous evaluation from a movement disorders neurologist. Admitting these cases into the archive based on nothing more than a screening form is counterproductive for mapping the neuropathology of ET. It is not ideal to accept donations with no phenotyping from an experienced movement disorders neurologist. The possibility of studying the changes in the brain of patients who have the wrong diagnosis is very high. Hence, a detailed screening process for thorough diagnostic confirmation is required.

#### B. Longitudinal follow-up of ET donors

In addition to a very detailed screening process, ET brain repositories require or benefit from a longitudinal follow up of donors. There are three important reasons that justify this approach: longevity of ET patients, changes in tremor phenotype over the years and lack of medical records assessing tremor.

First, although ET is associated with a slightly higher mortality rate than same-age healthy controls [78], ET patients appear to live longer than patients who suffer from other neurodegenerative diseases such as Alzheimer’s disease or PD [79,80]. One cannot predict the point of death, and this therefore often means that donors are followed for considerable time periods.

Second, the tremor in ET evolves over time. It is not static [35]. Furthermore, ET patients can develop incident PD [81]. Indeed, their risk for PD is elevated compared to that of age-matched controls [82]. Hence, longitudinal clinical follow up is needed to establish whether co-morbid PD has developed.

Third, not all ET patients see a physician to treat their tremor [25]. In a recent survey of 15,000 ET patients, only 42% were seeing a neurologist and, within that group, only 19% were seeing a movement disorders neurologist [25]. Furthermore, 26% relied on their primary care provider for tremor follow-up, whereas 28% did not see a provider for their disease [25]. These statistics indicate that a large number of ET patients do not have sufficient medical records to allow a thorough retrospective evaluation of their tremor. Furthermore, the complexity of ET and the academic debates regarding motor and non-motor features makes it difficult for primary care providers and general neurologists to accurately track the array of symptoms, affecting the quality of the available medical records. Anecdotally, our research team collects medical records to evaluate other comorbidities at the time of death and we have noticed obtaining information about the donor’s tremor is extremely difficult.

To meet the goals of our repository, the research team cannot rely on retrospectively-collected data. Designing a brain repository with a thorough prospective clinical schedule is pivotal and, as a result, our research team carefully evaluates participants over the years, and plans the donations with years or decades of anticipation. In an environment where questions become more specific and demanding, just procuring an ET brain is not enough – correct phenotyping and clinical correlation is important as well.

## Conclusion

In this manuscript, we explored three disease-specific aspects of the brain donation stages from an ET perspective: (1) elements influencing the donation decision, (2) involvement of family in the process and (3) factors influencing the decision for enrolling participants prospectively and evaluating them longitudinally.

We show that ET has a particular reality that affects the way we harvest brains. While some of the features we discuss are unique to ET, others may be features of brain banks centered on other diseases. The particular combination of features, though, is unique to ET. In the end, though, these comparisons are difficult to make as the published literature does not delve into disease specific factors that affect the brain donation process in disorders such as Alzheimer’s disease or PD. Indeed, little if anything has been written on *disease-specific factors* that influence the brain donation process. Sharing these perspectives is important for better understanding the background from which repositories operate. We encourage the brain repository community to share their own disease-specific elements influencing brain donations.
